# News and Coming Events

**Published:** 1909-09-11

**Authors:** 


### News and Coming Events.

The Royal Colleges of Physicians and Surgeons have acquired a site in Queen Square, Bloomsbury, London, W.C., for the new examination hall. The building on the Victoria Embankment has been sold to the Institution of Electrical Engineers.

The St. Bartholomew's Hospital Old Students' Dinner will be held on Friday, October 1, in the Great Hall, at 6.30 for 7 p.m. Dr. Herringham will be in the chair. Tickets, one guinea each, may be obtained from Mr. H. J. Waring, the Hon. Sec., or payment can be made at the door. The new Pathological Department will be open for inspection after the dinner.

Dr. H. D. Rolleston is orator for the day at the opening of the new session of the St. George's Hospital Medical School on October 1, 1909, at three o'clock, and will take for his subject "St. George's and the Progress of Physic." The oration will be followed by the annual meeting of the St. George's Club. The annual dinner will be held at the Prince's Restaurant in the evening, Mr. A. Marmaduke Sheild, F.R.C.S, consulting surgeon to the hospital, in the chair. Tickets may be obtained from the hon. secretaries, Medical School Dinner, St. George's Hospital, S.W.

In connection with the death of Dr. Whitelaw, of Kirkintilloch, the *Glasgow Medical Journal* tells the following interesting story of his early career: "Being called up one night, many years ago, he was walking along with the messenger, when he was set upon and knocked down in a lonely part of the road. His pockets were rifled, and he was left lying on the road, with a fracture of the fibula. One of the articles stolen was a clinical thermometer with which he had that evening taken the temperature of a patient suffering from enteric fever. He remembered the temperature registered, also that he had not shaken down the mercury before putting the thermometer back in his pocket, and he communicated these facts to the police. Some time afterwards a thermometer registering the identical temperature was discovered in a pawnshop in Glasgow, and by this means the police were enabled to track the doctor's assailants, and to arrest them in one of the Glasgow theatres."

Sir Stephen Mackenzie, F.R.C.P., M.R.C.S., died at the residence of his son at Dorking, Surrey, last week. Sir Stephen, who was a brother of the late Sir Morell Mackenzie, was born in 1844. He received his education at Christ's Hospital, the London Hospital, and at the Universities of Aberdeen and Berlin. He took his M.B. degree (highest honours and special honours for his graduate thesis) at Aberdeen in 1873, and his M.D. degree two years later. He became a member of the Royal College of Surgeons in 1869, and a member of the Royal College of Physicians in 1874; he was made a Fellow of the latter College in 1879. He was consulting physician to the London Hospital, the Poplar Hospital, and the Royal Ophthalmic Hospital, an ex-president of the Metropolitan Counties Branch of the British Medical Association, and was formerly Lecturer on Medicine and Pathology at the London Hospital Medical College. His publications include numerous articles in medical dictionaries and periodicals. Sir Stephen Mackenzie married in 1879 Helen, the youngest daughter of Mr. Benjamin Dulley, of Wellingborough, and had three sons and one daughter. He was knighted in 1903.

Dr. Robert Hazelton, of Oxford Gardens, North Kensington, W., and of Westland Road, Dublin, who died on June 29, aged 84, left estate of the gross value of £17,166 8s. 1d., of which the net personalty has been sworn at £17,085 5s. 11d.

Dr. Henry Radcliffe Crocker left estate valued at £17,778 gross, with net personalty £15,328. He bequeathed to the Medical School of University College Hospital his drawings, sketches, diagrams, atlases, and books relating to diseases of the skin.

The Medical Officer of Health for the borough of Southwark in his annual report states that the estimated population of Southwark at the middle of 1908 was 210,442. The death-rate was 16.3 per 1,000, as against 18.0 in 1907. The birth-rate, 28.1 per 1,000, was the lowest in the history of the borough. In 1900 it was 33.3 per 1,000. The infantile mortality was 13.1 per 1,000 births, the lowest figure ever recorded; much of this has been brought about by the disposition at the present time of mothers to breast-feed their children and by the Notification of Births Act, 1907. Of infectious diseases cerebro-spinal fever proved fatal in six cases. The number of cases of diphtheria was the smallest on record, and there was also a considerable reduction in the mortality from tuberculosis. The mortality is, however, still very high. The deaths from phthisis were 414.

Dr. Paul Dorveaux has written a monograph on the history and employment of "silkworm-gut." The first mention of the use of the substance (which is really the "sericigenous organ" or silk-spinning tube of the silkworm) in Europe is to be found in Sir John Hawkins' (1760) edition of Walton and Cotton's "Compleat Angler" (Chapter XXI). It was then a novelty, just brought from China. In most European languages it is identified by a literal translation of the English title (*Seidenwurmdarm, intestino de gusano de seda*, etc.). Gariot, who wrote on buccal maladies (Paris, 1805), recommends silkworm-gut for dental prothesis, and in 1813 George Fielding published, in the "Transactions of the Medico-Chirurgical Society of Edinburgh," an article "On the Use of a New Substance—Silkworm-gut—for Securing Divided Arteries." He was in search of a ligature "likely to be absorbed or dissolved in the animal fluids," when his assistant, E. Heseltine, made this suggestion to him. James Wardrop, another British surgeon, used it a few years later (1828) for the ligature of the carotid artery. In 1844 the "poil de Messine" or "crin de Florence" (other French names for the same substance) figures for the first time in the French Customs tariff, and "since Pasteur and Lister's discoveries" this article "at last occupies the prominent place which Fielding and Passavant had dreamed of giving it for surgical purposes." In 1891 it was mentioned in the "Real-Encyclopedie der gesammten Pharmacie," in 1893 in Dorvault's "Officine," and in 1906 in Martindale and Westcott's "Extra Pharmacopœia." Sir Robert Hart briefly alluded to the Chinese method of preparation in his description of the Ningpo exhibit at the Berlin Fishery Exhibition, 1880. China, Italy, and Spain are still the principal sources of supply for this substance. Dr. Dorveaux is well known as the distinguished librarian of the Paris Superior School of Pharmacy, and his monograph on silkworm-gut is a good instance of his erudition and very painstaking research.

Special precautions against the introduction of cholera are being taken by the medical officer at Hull. A notice has been issued stating that all tourists and passengers arriving from Rotterdam would be medically examined.

The annual dinner of the Society of Medical Officers of Health will take place at the Criterion Restaurant, Piccadilly, W., on Friday, October 8, 1909. Ladies are to be invited as guests. Early application for tickets is desirable to Dr. Priestley, Lambeth Town Hall, Brixton Hill, S.W. (single, 7s. 6d.; double, 15s.—without wine).

Mr. H. Colborne, M.R.C.S., who for 30 years has rendered valuable service as borough meteorologist of Hastings, was recently the recipient of a valuable marine chronometer, together with a cheque. The chronometer bore the following inscription: "Presented to H. Colborne, Esq., M.R.C.S., in appreciation of 30 years' service as Borough Meteorologist of Hastings, August, 1909."

The death is announced of Dr. John Bowes, who had practised at Herne Bay for the last 50 years. Dr. Bowes entered Guy's Hospital as a student in the early 'fifties, and afterwards became a member of the Royal College of Surgeons and a Licentiate of the Royal College of Physicians. He was an ex-president of the East Kent and Canterbury Medical Society, a Fellow of the Zoological Society, and a justice of the peace for the county of Kent. He was 75 years of age.

The total number of deaths from cholera at Rotterdam since August 20 is now eleven, and there are three new cases. All the cases have been caused by the use of impure river water. The total number of patients is 11, and 90 persons are under observation. The militia, who were summoned for their usual drill, have now received orders to remain at home. There have also been two deaths at Gorinchem and Arnhem.

The Polyclinic and Medical Graduates' College, Chenies Street, W.C., will open for the Autumn Session on Monday, the 13th inst. The usual afternoon clinics will commence on the day following, and will be continued according to the plan followed in previous sessions. A special vacation session of practical classes has been arranged to extend from Monday, September 13, to Friday, October 1. Entries for these classes should be made at an early date. Practitioners who join the College as subscribers on or after the 13th inst. are free from all further subscription until January 1, 1911. A complete programme of the Autumn Session is now ready, and can be obtained on application to the Medical Superintendent, 22 Chenies Street, W.C.

The death was announced on August 22 of Dr. Radcliffe Crocker, F.R.C.P., at Engelberg, Switzerland. Dr. Crocker was an honorary member of the American, French, Austrian, German, and Italian Dermatological Societies, physician to the skin department of University College Hospital, and a Vice-President of the British Medical Association. He revised the Dermatological Catalogue which is in use at the Museum of the Royal College of Surgeons at the special request of the Council, whilst his many publications include valuable additions to the standard works on skin diseases, chief among which may be noted a treatise which ran into the third edition, and an atlas on the same subject. Dr. Crocker married in 1880 the daughter of Dr. Fussell, of Brighton, who survives him.

An action brought in Edinburgh against the Edinburgh Hospital and Dispensary for Women and Children, to recover £100 damages in respect of injury through being burned on the leg by a hot-water bottle while under the influence of an anæsthetic in the said hospital, has resulted in the claimant being awarded £25 damages.

The resignation by Mr. N. W. Bourns, M.R.C.S., L.R.C.P., is announced of his post as Anæsthetist to the Cancer Hospital, Fulham Road. Mr. Bourns has held this appointment for twenty-six years, and his connection with the institution as House Surgeon and Registrar dates back even further still.

Michael Servetus who was martyred by Calvin, is to have a statue at Vienna in Dauphine in the Department of Isere. Servetus was, perhaps, the first to describe, more or less accurately, the lesser circulation. His book which contains the theory was published at Vienna in 1543. It is entitled "Christianisimi restitutio," and, like his "heretical" work "De trinitate erroribus," is a bibliographical rarity.

The Humbert I. prize, for the best treatise on or best discovery in the realm of orthopædics offered by the Instituto Rizzoli, Bologna, is now open to competition. The prize is of the value of 3,500 lire, and the competition is open to all qualified men. Particulars may be had on application to the Director of the Instituto Rizzoli, G. Bacchelli, S. Michele in Bosco, Bologna.

M. Clemenceau, the former French Prime Minister and a retired member of the medical profession, is credited with the intention of producing this autumn a play founded on his novel, *Les Plus Forts*, which was published ten years ago. According to various journals, M. Tarride, who is taking over the Renaissance Theatre from M. Guitry, will produce the play.

On August 26 the mayor of Rotterdam made a reassuring statement in the Town Council with regard to the outbreak of cholera. No further deaths have occurred, but over forty people are under observation. It is supposed that the cholera was caused by infected ballast or tank-water taken in by timber-ships at Riga, St. Petersburg, or Kronstadt, and discharged by them at the mouth of the Meuse before being disinfected at the port of Rotterdam.

In the House of Commons Captain Craig asked the Secretary of State for the Home Department whether his attention had been called to the increase in recent years of the sale of quack medicines; whether he was aware that it had been proved that such nostrums frequently contained nothing but harmless drugs, coloured grease, coloured water, small quantities of aloes, pilules of sugar, etc., though advertised to cure a multitude of different maladies; whether he was aware that the chief cost of such quack medicines was in the advertising; and whether he would appoint a small Commission to inquire into and report upon the whole subject? Mr. Secretary Gladstone, in a written reply, said: I understand that inquiries are being made, at the instance of the Lord President of the Council, as to whether the practice of medicine by unqualified persons is extending, and as to the effects produced by such practice. These inquiries will no doubt throw some light on the question of the use of quack medicines, and I think it will be advisable to await their result.

